# Global Changes of 5-mC/5h-mC Ratio and Methylation of Adiponectin and Leptin Gene in Placenta Depending on Mode of Delivery

**DOI:** 10.3390/ijms22063195

**Published:** 2021-03-21

**Authors:** Aneta Słabuszewska-Jóźwiak, Marcelina Malinowska, Anna Kloska, Joanna Jakóbkiewicz-Banecka, Mariusz Gujski, Iwona Bojar, Dorota Raczkiewicz, Grzegorz Jakiel

**Affiliations:** 1 First Department of Obstetrics and Gynaecology, Centre of Postgraduate Medical Education, Żelazna 90, 01-004 Warsaw, Poland; grzegorz.jakiel1@o2.pl; 2 Department of Medical Biology and Genetics, Faculty of Biology University of Gdańsk, Wita Stwosza 59, 80-308 Gdańsk, Poland; marcelina.malinowska@ug.edu.pl (M.M.); anna.kloska@ug.edu.pl (A.K.); joanna.jakobkiewicz-banecka@ug.edu.pl (J.J.-B.); 3 Department of Public Health, Medical University of Warsaw, Nielubowicza 5, 02-097 Warsaw, Poland; mariusz.gujski@doktor.pl; 4 Department of Women’s Health, Institute of Rural Health in Lublin, Jaczewskiego 2, 20-950 Lublin, Poland; iwonabojar75@gmail.com; 5 Department of Medical Statistics, School of Public Health, Center of Postgraduate Medical Education, Kleczewska 61/63, 01-826 Warsaw, Poland; dorota.bartosinska@gmail.com

**Keywords:** cesarean section, adipokines, DNA methylation, 5-mC/5-hmC ratio, *ADIPOQ*, *LEP*, placenta

## Abstract

It was suggested that the epigenetic alterations of the placenta are associated with obesity, as well as the delivery mode. This study aimed to assess the effect of maternal outcome and delivery procedure on global placental DNA methylation status, as well as selected 5’-Cytosine-phosphate-Guanine-3’ (CpG) sites in *ADIPOQ* and *LEP* genes. Global DNA methylation profile in the placenta was assessed using the 5-methylcytosine (5-mC) and 5-hydroxymethylcytosine (5-hmC) ratio evaluated with the ELISA, followed by target gene methylation patterns at selected gene regions which were determined using methylation-specific qPCR in 70 placentas from healthy, pregnant women with single pregnancy. We found no statistically significant differences in 5-mC/5-hmC ratio between intrapartum cesarean sections (CS) and vaginal deliveries (*p* = 0.214), as well as between elective cesarean sections and vaginal deliveries (*p* = 0.221). In intrapartum cesarean sections, the *ADIPOQ* demethylation index was significantly higher (the average: 1.75) compared to elective cesarean section (the average: 1.23, *p* = 0.010) and vaginal deliveries (the average: 1.23, *p* = 0.011). The *LEP* demethylation index did not significantly differ among elective CS, intrapartum CS, and vaginal delivery groups. The demethylation index of *ADIPOQ* correlated negatively with *LEP* in the placenta in the vaginal delivery group (*r* = −0.456, *p* = 0.017), but not with the global methylation. The methylation of a singular locus might be different depending on the mode of delivery and uterine contractions. Further studies should be conducted with locus-specific analysis of the whole genome to detect the methylation index of specific genes involved in metabolism.

## 1. Introduction

Currently, a cesarean section is the most common procedure in obstetrics and gynecology [1]. According to a recent meta-analysis, children born via cesarean section are at a higher risk of developing obesity in childhood [2,3]. This may be caused by the imbalance in the metabolic homeostasis of adipokines, i.e., cytokines which are mainly produced by white adipose tissue (WAT) in the course of pro- or anti-inflammatory activities [4]. Adiponectin and leptin are the two best-studied peptide hormones secreted into the circulation, where they function as messengers between the adipose tissue and other structures, including the placenta [5].

During pregnancy, leptin is produced in the adipocytes of both the pregnant woman and the fetus. The synthesis and the secretion of this hormone, as well as the presence of leptin receptors, were described within trophoblast cells [6,7]. The placental hormone molecule does not differ from leptin produced in the adipose tissue. It was suggested that leptin was involved in the normal development of the fetus, influencing its growth, proper angiogenesis, hematopoiesis, and the development of the immune and central nervous systems [8]. Furthermore, leptin is involved in trophoblast implantation and placental development [9], stimulating proliferation, survival, and the invasion of trophoblast cells [10]. In addition, leptin level in blood serum usually correlates with the amount of the adipose tissue and with body mass index (BMI) [11], although no differences in the concentrations of placental leptin were observed between obese and slim women [12].

**Figure 1:**
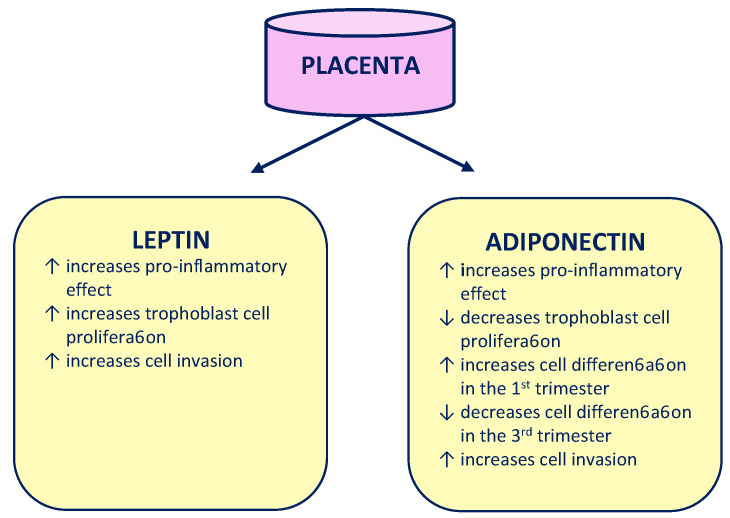
Role of leptin and adiponectin in the placenta.

Adiponectin, similarly to leptin, has endo- and paracrine effects in the placenta. This hormone secreted by trophoblast cells has a pro-inflammatory effect, which is mainly manifested in the third trimester of pregnancy [13]. At the beginning of pregnancy, adiponectin stimulates the secretion of hCG and leptin by syncytiotrophoblast cells, increasing chorion proliferation. These processes become slower in the third trimester of pregnancy [14]. However, it does not affect the morphology of the placenta at the term of delivery. Similar to leptin, adiponectin promotes the invasion of trophoblast into the temporally altered endometrial mucosa [15]. The role of leptin and adiponectin in the placenta is presented in Figure 1.

The placenta is a unique and complex organ responsible for intragestational fetal–maternal interaction. It plays a crucial role in delivering water, oxygen, nutrients, and growth-regulating signals from the mother to the fetus. In addition, there is some evidence that placental global methylation changes may limit its function and have an impact on fetal development [16,17,18]. DNA methylation is the addition of a methyl group at the carbon-5 position of cytosine catalyzed by DNA methyltransferases (DNMTs), mainly in a 5’-Cytosine-phosphate-Guanine-3’ (CpG) sequence. Methylation can change the properties of the sequence, i.e., when located in the gene promoter, it represses gene transcription [19,20]. Methylation changes in the placenta caused by environment-related factors (e.g., diet) and endogenous factors (e.g., inflammation, hypoxia) may trigger changes in the expression of genes that are important for fetal homeostasis. Furthermore, the type of delivery contributes to changes in the global methylation of the placenta [21]. Conversely, the detection of global DNA methylation may provide only preliminary information related to changes in total methylcytosines in CpG islands and undetected locus-specific and simple gene changes.

In the present study, we investigate the impact of maternal outcome and delivery procedure on the global placental DNA methylation status, as well as on the methylation of selected CpG sites in adiponectin and leptin gene sequences.

## 2. Results

### 2.1. Characteristics of the Study Group

Table 1 presents study group characteristics comprising the following elements: maternal and gestational ages, parity, pre- and intragestational BMI, weight gain, weight and gender of the newborn, both 1 and 5 min Apgar scores, type of analgesia administered in three modes of delivery, and the duration of uterine contractions in the case of vaginal deliveries. Significant differences were noted with regard to the maternal and gestational age among the elective cesarean section, intrapartum cesarean section (CS), and vaginal delivery groups. Parity, pre- and intragestational body mass index (BMI), intragestational weight gain, weight and gender of the newborn, and 1 and 5 min Apgar scores were not significantly different with regard to the elective CS, intrapartum CS and vaginal delivery groups. The duration of uterine contractions ranged from 1 to 12 h in vaginal deliveries (the average: 5.17 h). All women undergoing cesarean sections were administered spinal analgesia, while epidural analgesia was administered in 59.26% of vaginal delivery cases. Neither inhalation nor intravenous analgesia was administered in the vaginal delivery group.

### 2.2. 5-methylcytosine (5-mC) to 5-hydroxymethylcytosine (5-hmC) Ratio in the Placenta in the Study Group

**Figure 2:**
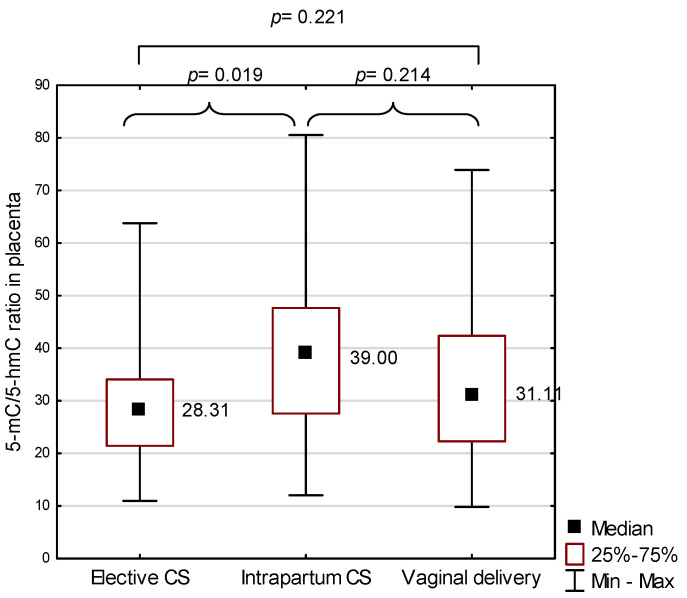
The 5-methylcytosine to 5-hydroxymethylcytosine (5-mC/5-hmC)ratio in the placenta against the mode of birth; *p* represents results from the Mann–Whitney U test to compare 5-mC/5-hmC ratio in the placenta between two modes of birth. CS—cesarean section.

The global DNA methylation profile in the placenta was estimated by the measurement of 5-mC and 5-hmC levels and presented as their ratio. The elective cesarean section group was characterized by a significantly lower 5-mC/5-hmC ratio in the placenta (average: 28.31) in comparison with the intrapartum CS group (average: 39.00, *p* = 0.019). No significant differences were noted in the 5-mC/5-hmC ratio in the placenta between intrapartum CS and vaginal delivery groups (*p* = 0.214), as well as between elective CS and vaginal delivery groups (*p* = 0.221; Figure 2).

A positive correlation occurred between 5-mC/5-hmC ratio and the weight gain during pregnancy expressed in kg (*r* = 0.238, *p* = 0.047). The 5-mC/5-hmC ratio did not correlate with the maternal and gestational age, parity, pre- and intragestational BMI, newborn weight, or 1 and 5 min Apgar scores in all modes of delivery (Table 2).

Male newborns had a significantly higher 5-mC/5hmC ratio in the placenta (average: 44.45) than female newborns (average: 27.57) in the intrapartum cesarean section group (*p* = 0.028). Gender differences were not observed in the elective cesarean section or in vaginal delivery group (*p* = 0.346 and *p* = 0.657, respectively). No significant differences were noted between the 5-mC/5-hmC ratio in women with and without epidural analgesia (*p* = 0.622). Moreover, the ratio did not correlate with the duration of uterine contractions in the vaginal delivery group (*r* = 0.160, *p =* 0.426).

### 2.3. ADIPOQ in the Study Group

**Figure 3:**
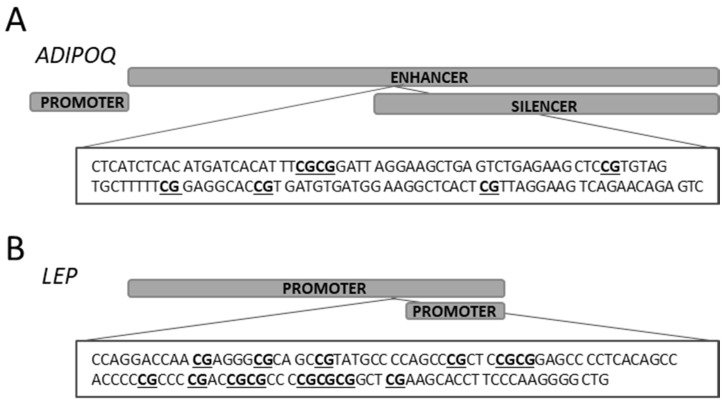
5’-Cytosine-phosphate-Guanine-3’ (CpG) sites selected for methylation-specific quantitative PCR (MS-qPCR). Chromosomal localization and nucleotide sequence in human-based *ADIPOQ* reference sequence (NC_000003.12) with six CpG sites (**A**) and *LEP* (NC_000007.14) reference sequence (NC_000007.14) (**B**).

Using the MethPrimer online tool, we selected regions with CpG sites for determination of the methylation status of the *ADIPOQ* and *LEP* genes [22]. In order to look for CpG islands in the *ADIPOQ* gene, the 4830 bp sequence including a promoter and a large fragment of the enhancer and silencer region located within intron 1 was taken for analysis. Two predicted CpG islands were found in the analyzed sequence (1: 185 bp (3087–3271); 2: 108 bp (3940–4047)). The *ADIPOQ* gene methylation status in genomic DNA isolated from the placenta was determined on the second CpG island located downstream from the promoter sequence and within the enhancer, whereby the analyzed region contained six CpG sites located in the first intron (Figure 3A). Validated primer sequences and specification used for the methylation-specific quantitative PCR (MS-qPCR) assay are listed in Table 3.

**Figure 4:**
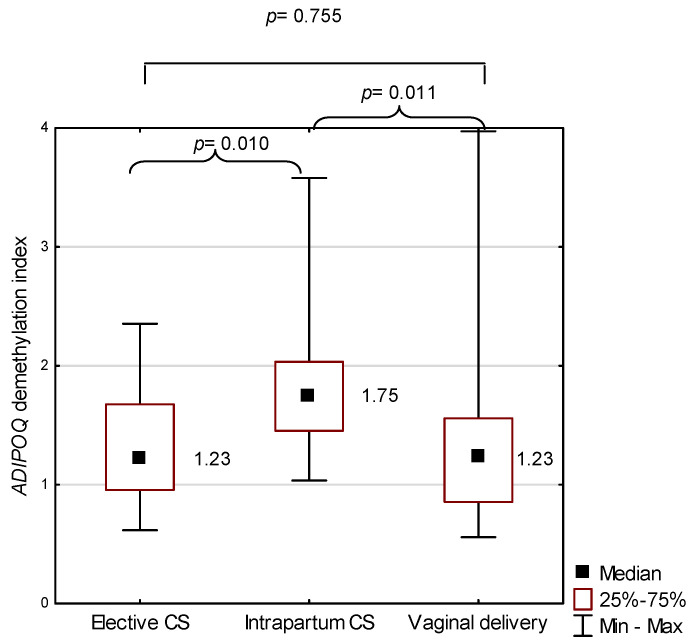
*ADIPOQ* demethylation index against the mode of birth; *p* represents results from the Mann–Whitney U test to compare *ADIPOQ* demethylation index between two modes of birth. CS—cesarean section.

In the intrapartum cesarean section group, the demethylation index for *ADIPOQ* in the placenta was significantly higher (the average: 1.75) in relation to the elective cesarean section group (average: 1.23, *p* = 0.010), and in comparison with the vaginal delivery group (average: 1.23, *p* = 0.011). The *ADIPOQ* demethylation index in the placenta was not significantly different between the elective CS and the vaginal delivery groups (*p* = 0.755; Figure 4).

A negative correlation was found between *ADIPOQ* demethylation index in the placenta and the parity (*r* = −0.40, *p* = 0.044). *ADIPOQ* demethylation index was not correlated with maternal and gestational ages, pre- and intragestational BMI, intragestational weight gain, newborn weight, or 1 and 5 min Apgar score in every mode of delivery (Table 4).

The *ADIPOQ* demethylation index in the placenta was significantly higher in the case of female newborns (average: 1.36) than in the case of male newborns (average: 0.86) in the vaginal delivery group (*p* = 0.025). Such a correlation did not occur in the elective or intrapartum CS groups (*p* = 0.884 and *p* = 0.999, respectively).

The *ADIPOQ* methylation in the placenta was nearly significantly higher in women with epidural analgesia (the average 1.31) than in women without such analgesia (0.86 on average, *p* = 0.057). *ADIPOQ* did not correlate with the duration of uterine contractions in the case of vaginal deliveries.

### 2.4. LEP in the Study Group

The sequence analysis of the *LEP* gene revealed the presence of two promoter regions. One CpG island was found within the longer promoter sequence, and this region with 14 CpG sites was taken for analysis (Figure 3B).

**Figure 5:**
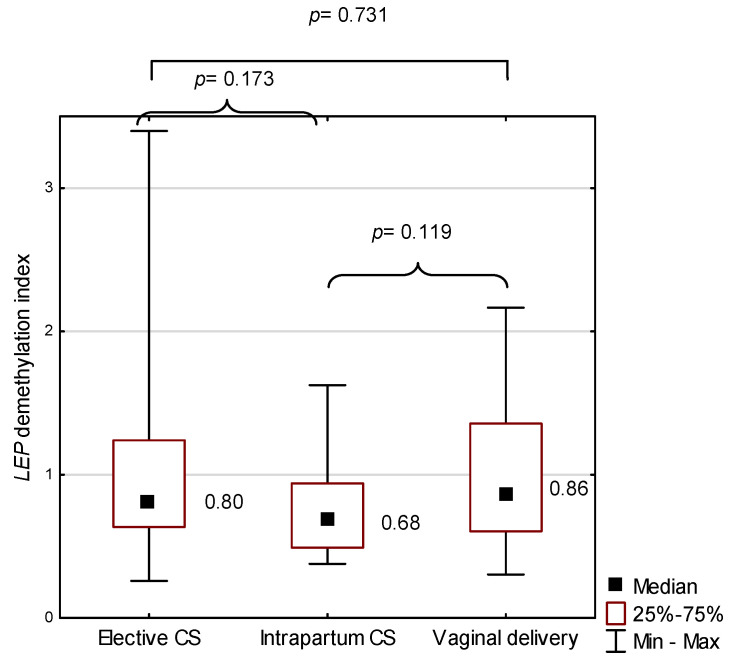
*LEP* demethylation index against the mode of birth; *p* represents results from the Mann–Whitney U test to compare *LEP* demethylation index between two modes of birth. CS—cesarean section.

*LEP* demethylation in placenta did not significantly differ among elective CS, intrapartum CS, and vaginal delivery groups (*p* > 0.05; Figure 5).

A positive correlation was found between *LEP* demethylation in the placenta and parity in the vaginal delivery group (*r* = 0.606; *p* = 0.001). *LEP* demethylation index was not correlated with the maternal and gestational ages, pre- and intragestational BMI, intragestational weight gain, newborn weight and gender, or 1 and 5 min Apgar score in every mode of delivery (Table 5).

*LEP* demethylation index in the placenta was significantly lower in women with epidural analgesia (average: 0.64) than in women without such analgesia (average: 1.21, *p* = 0.025). A significant negative correlation was revealed between *LEP* methylation and duration of uterine contractions (*r* = −0.395; *p* = 0.041).

### 2.5. Mutual Correlations between Global Locus-Specific Methylation Status for ADIPOQ and LEP Demethylation Index in the Study Group

*ADIPOQ* demethylation index in the placenta correlated negatively with *LEP* demethylation index in the placenta in the vaginal delivery group (*r* = −0.456, *p* = 0.017). However, 5-mC/5-hmC ratio did not correlate with *ADIPOQ* and *LEP* demethylation indices in the different modes of delivery (Table 6).

## 3. Discussion

The human genome is highly methylated (>70%). However, the placenta is usually hypomethylated [23]. RNA-seq analysis demonstrated that genes in partially methylated domains, constituting up to 40% of the placental genome, were repressed, and the possibility of their transcription into proteins was lower [24]. The Epigenetic Impact of Childbirth research collaboration stated that the intrapartum use of synthetic oxytocin, antibiotics, or cesarean section exerted some influence on the neonatal epigenome remodeling processes, resulting in health consequences in the offspring [25]. The hypothesis has not been fully elucidated, and data to justify the hypothesis are insufficient. We previously reported widespread changes in placental global DNA methylation depending on the mode of delivery [26]. The present study includes an extension of the analysis to 5-mC/5-hmC ratio and the levels of DNA methylation in the promoter region of *LEP* and *ADIPOQ* genes in the placental tissue after different modes of delivery. We found a significantly lower 5-mC/5-hmC ratio in the elective cesarean section group compared to intrapartum cesarean section (*p* = 0.019). It is known that 5-hmC is an epigenetic modification which is of importance in the course of embryogenesis. However, data are scarce on its physiological role and predictors. The distribution of 5-hmC was demonstrated to be a relatively stable epigenetic mark with regard to in vitro and animal models. It was not only present as a transient product of demethylation, thus possibly playing an independent regulatory function role [27]. Several studies were performed to examine fetal 5-hmC disruption during human development, e.g., preeclampsia and gestational diabetes [28]. Such factors impact the fetus for a longer perinatal period than uterine contractions or a surgical procedure, even though 5-mC/5-hmC ratio is higher when uterine contractions appear. Schlinzing et al. demonstrated that the global DNA methylation in white blood cells sampled from umbilical cord blood was elevated in newborns delivered via cesarean section without labor compared to those who were born vaginally [29]. Franz et al. found no difference in the global methylation between newborns in the vaginal delivery group compared to the CS group. They claimed that only single-gene methylation was markedly higher in newborns delivered via elective CS. Moreover, hypermethylation was noted in case of *FOXP3*, *CD7*, *ELA2*, and *IRF1*, while only *ELA2* and *IRF1* were associated with markedly higher hypermethylation in the CS group [30]. The results presented in this study showed significantly higher placental *ADIPOQ* promoter methylation in the intrapartum cesarean section group, while the methylation of *LEP* promoter was the lowest in this group, but the difference was not significant. According to these findings, the upregulation of *ADIPOQ* gene expression might be attributed to the duration of uterine contractions. A reported significant methylation of three CpG islands at the placental *ADIPOQ* locus and lower levels of DNA methylation in the *ADIPOQ* gene promoter in the fetus were reported by Bouchard et al. The results revealed a correlation of lower DNA methylation levels of the *ADIPOQ* gene with higher glucose levels in the mother during the second trimester of pregnancy [31]. Furthermore, it was reported that maternal obesity (no concomitant gestational diabetes) was linked to the increased DNA methylation of the leptin promoter only in the fetus and to adiponectin promoter hypomethylation. The authors concluded that the leptin and adiponectin systems (i.e., ligands and receptors) were downregulated in the placenta in obese pregnant women in comparison with nonobese pregnant women [32]. None of these studies described the type of delivery. Perhaps a long-term factor is necessary to change the placental methylation of promoters in the above genes. Global DNA methylation in the human placenta was more abundant in obese relative to slim pregnant women [33]. Another study found increased epigenetic alterations of transcription regulators in the placenta of women with inadequate gestational weight gain compared to women with normal weight gain during pregnancy [34]. These placental epigenetic changes due to long-term factors such as an increase in maternal BMI [35] or longitudinal stress, but not the type of delivery, may explain the intrauterine mechanisms of the developmental triggers of obesity in the offspring.

The adiponectin gene is regulated by DNA methylation, an epigenetic factor that is influenced by both genetic and environmental factors [31,36,37], which may add further complexity to adiponectin regulation during pregnancy. In general, increased methylation is frequently deemed to be related to the repression of gene transcription. However, the complexity of the underlying mechanisms indicates that it is associated with genomic/genetic location [38]. Adiponectin concentrations during pregnancy are affected by body mass index [39]. Our study demonstrated that pregestational BMI, intragestational BMI, intragestational weight gain, and newborn weight were not correlated with adiponectin methylation in the promotor region. A significant negative correlation was shown between parity and *ADIPOQ* methylation in the placenta (*r* = −0.240, *p* = 0.044). However, a positive correlation occurred between *LEP* and parity in the group delivering vaginally (*r* = 0.606, *p* = 0.001).

Leptin is responsible for the activation of pro-inflammatory cytokine and prostaglandin release from human placental explants. Its action involves the modulation of endocrine placental function [40]. The dysregulation of placental leptin was suggested in the pathogenesis of numerous pregnancy-related pathologies, including gestational diabetes, recurrent miscarriage, intrauterine growth retardation, and preeclampsia [41]. Our study was carried out in healthy women with term pregnancies, and no correlation was present between maternal age, pregestational BMI, intragestational weight gain, or newborn weight and *LEP* promotor region methylation. Although *LEP* methylation in the placenta was not significantly different among the three modes of delivery (*p* > 0.05), a significant negative correlation was noted with the duration of uterine contractions and epidural analgesia in the vaginal group. This means that the intrauterine stress of uterine contractions and maternal pain did not change the placental 5-mC/5-hmC ratio significantly, but it might influence the methylation of a single gene.

Sexual dimorphism in adiponectin regulation was observed with higher levels of molecular weight (HMW) expressed in women compared to men [5]. In our study, *ADIPOQ* methylation in the placenta was significantly higher in the case of female newborns (average: 1.36) than in male newborns (average: 0.86) in the vaginal delivery group (*p* = 0.025). However, it was not observed in the elective or intrapartum cesarean section group. In contrast to *ADIPOQ* methylation, *LEP* methylation did not correlate with newborn gender. Mansel et al. presented limited evidence regarding the sex-specific relationship among early-life *LEP* gene methylation, weight, and severe obesity in 4 year old individuals. This might be related to sex differences in signaling associated with the regulation of metabolism in the hypothalamus and higher overweight and obesity rates in the female population [42].

To our knowledge, this study is the first placental epigenetic investigation of correlations between DNA methylation levels in the promotor of placental *LEP* and *ADIPOQ* and the type of delivery. Our study revealed a significant negative correlation between *LEP* methylation and the duration of uterine contractions (*r* = −0.395; *p* = 0.041). Opposing effects are exerted by leptin and adiponectin on placental amino-acid uptake at term. Therefore, those two adipokines may control nutrient delivery to the fetus through the placenta [43]. However, the majority of studies investigating the leptin and adiponectin systems in the human placenta were performed in women with gestational diabetes. In our study *ADIPOQ* gene methylation correlated negatively with *LEP* gene sequence methylation patterns in the placenta in the vaginal delivery group (*r* = −0.456, *p* = 0.017).

In our study, differences in the global methylation status in the placenta were observed depending on the delivery mode. With regard to the elective cesarean section group, the global methylation status was significantly lower compared to intrapartum cesarean section, but *LEP* and *ADIPOQ* demethylation indices did not significantly differ between the elective cesarean section and vaginal delivery groups. Therefore, it is necessary to conduct locus-specific analysis of the whole genome to detect changes of the methylation status of specific genes engaged in fetal metabolic processes. Such an analysis might explain the occurrence of obesity in children delivered via cesarean section.

A limitation of the present study may be related to the absence of the determination of perinatal maternal leptin and adiponectin concentration and the absence of comparing *ADIPOQ* and *LEP* expression with mRNA expression and methylation levels. Literature data showed that the placenta seemed not to be a key source tissue of adiponectin [44]. Subsequent cross-tissue studies may result in obtaining additional data on whether and where *ADIPOQ* gene sequence methylation patterns undergo alterations from the perspective of hyperglycemic maternal–fetal conditions. The small sample in which a cesarean section was preceded by regular uterine contractions is insufficient to fully confirm the influence of the mode of delivery on DNA methylation of single genes.

## 4. Materials and Methods

### 4.1. Study Group

This study was conducted in the First Department of Obstetrics and Gynecology, Center of Postgraduate Medical Education in Warsaw between July 2016 and January 2017 and included 70 placentas of Caucasian newborns from singleton term (gestational age ≥ 37 weeks) pregnancies.

On the day of hospitalization, the pregnant women completed a survey and an anthropometric questionnaire, and they provided their written consent to participate in the study. Information regarding the height, pregestational body weight, intragestational weight gain, and course of their pregnancy was obtained from the medical documentation. Exclusion criteria for the study were as follows: multiple pregnancies, preterm birth (birth < 37 weeks), infant weight below 2500 g, and Apgar score below 7 points at 1 min and 5 min after birth or on admission to the Neonatal Intensive Care Unit within the first 24 h postnatally. Children with inborn defects or chromosomal abnormalities were also excluded from the study. Maternal age below 18 years or diseases such as diabetes (diagnosed pre- or intragestationally) or arterial hypertension, as well as heart disorders (diagnosed pre- or intragestationally), cholestasis or hepatic pathologies, renal insufficiency, or respiratory diseases including asthma, pulmonary disorders, or pulmonary embolism and psychiatric diseases were also excluded from the protocol. Women hospitalized during pregnancy due to bleeding from the genital tract or hospitalized because of preterm birth with a cervical cerclage, pessary, or PROM (preterm rupture of membranes) were excluded from the study. Taking drugs such as glucocorticosteroids or antibiotics less than 2 weeks before hospitalization, cigarette smoking, alcohol drinking, or placental malformation were also exclusion criteria.

A total of 30 placentas were delivered during elective cesarean section due to the fetal preterm breech presentation, tokophobia, or a previous maternal cesarean section. In 13 cases, cesarean section was followed by regular uterine constriction. None of the cesarean sections were performed due to fetal distress or prolonged labor, or with general analgesia. The control group was composed of 27 placentas delivered after vaginal delivery. Neither vacuum extractor nor obstetric forceps was used during vaginal deliveries. No delivery was preceded by oxytocin administration in order to induce labor, the administration of prostaglandins, or the premature rupture of membranes.

In all cases, placenta delivery was natural, and the tissue was collected within 45 min. The placenta was excluded from the study in the case of placental tissue defects or if it was manually extracted. Full-thickness placental tissue fragments were obtained adhering to the aseptic technique with the use of sterile instruments. All tissue samples were retrieved in the proximity of the umbilical cord insertion site to the placenta. The tissue was separated into three horizontal fragments from the basal toward the chorionic surface. Each sample was thoroughly rinsed in a cold sterile saline solution (Phosphate Buffered Saline, pH 7.5). Samples obtained from different sections were snap-frozen in liquid nitrogen and stored at 80° C for the analysis.

The study protocol was approved in 29 January 2014 by the Ethics Committee of the Postgraduate Medical Education Center in Warsaw (No. 8/PB/2014).

### 4.2. DNA Extraction and Sodium Bisulfite Conversion

Genomic DNA was isolated from placenta samples with the use of DNeasy^®^ Blood and Tissue Kit (Qiagen, Hilden, Germany) in compliance with the manufacturer’s protocol. Briefly, about 20 mg of each placenta sample was cut into small pieces and chemically digested with proteinase K at 56 °C until completely lysed. After protein digestion, DNA was precipitated in 200 µL of ethanol, washed, and resuspended in 150 µL of Tris-EDTA (TE) buffer. Then, it was stored at −20 °C. During isolation, DNA was treated with RNaseA (Blirt, Gdansk, Poland). DNA concentration and quality were estimated using a NanoDrop ND-1000 spectrophotometer (NanoDrop Technologies, Inc., Wilmington, DE, USA). Bisulfite DNA conversion was carried out with the use of the EpiJET Bisulfite Conversion Kit (Thermo Scientific, Valtham, MA, USA). Briefly, an aliquot of 500 ng of input DNA was used for bisulfite conversion according to the manufacturer’s protocol, followed by a clean-up procedure with spin columns provided in the kit. The concentration of bisulfite modified DNA samples was measured with the NanoDrop ND-1000 spectrophotometer using a value of 40 μg/mL for absorbance at 260 nm = 1.0. The concentration of bisulfite converted DNA samples was adjusted to 10 ng/μL prior to downstream analysis. Converted DNA was used as a template in methylation-specific real-time qPCR analysis (MS-qPCR).

### 4.3. Methylation-Specific Real-Time qPCR

Primers specific for methylated and unmethylated target DNA for real-time qPCR experiments were designed using the MethPrimer online tool [22]. First, the input DNA sequences of target genes were analyzed for potential CpG islands, and primers were designed around the predicted CpG islands or user-defined CpG sites. Primers were synthetized by ThermoFisher (Glasgow, UK); their sequences and characteristics are listed in Table 3.

Real-time qPCR amplification was carried out in a total volume of 10 µL with the use of the SG qPCR Master Mix (2X) kit (EurX, Gdańsk, Poland) with a final 0.5 µM concentration of each primer and 10 ng of bisulfite-modified DNA template. Reactions were run on the LightCycler^®^ 480 Instrument II (Roche Diagnostics Ltd., Rotkreuz, Switzerland) in the following conditions: 95 °C for 2 min (initial denaturation), followed by 40 cycles of 94 °C for 15 s, 57–60 °C (depending on the primer pair used) for 30 s, and 72 °C for 30 s for *ADIPOQ* and *LEP* targets. Melting curve analysis was performed in the range from 65 °C to 95 °C. Crossing threshold (Ct) values generated by the LightCycler^®^ 480 Software were between 26 and 30 cycles. Reactions with no template were used in each run to control the purity of the reagents. Reactions with nonmodified genomic DNA were used to assess the primer specificity to the bisulfite-modified DNA template only. All reactions were run in duplicate, and the Ct values obtained were averaged. Primers were validated for amplification efficiency using twofold dilutions of bisulfite-modified DNA as a template; the efficiency was estimated between 80% and 100%, and only primers with similar efficiencies (difference below 10%) obtained for methylated and demethylated target sequences were used as a set for subsequent qPCR experiments. The demethylation index (DMI) was calculated from the mean Ct values obtained for methylated and unmethylated DNA templates according to the method developed by Akirav et al. [45],

$DMI=2^{\left(methylated\;C_{t}\;-\;demethylated\;C_{t}\right)}$.

The demethylation index describes the relative abundance of demethylated DNA; DMI <1 indicates that the sequence is predominantly methylated, while DMI >1 indicates that the sequence is predominantly demethylated. 

### 4.4. Global DNA Methylation Status

The methylation status of genomic DNA extracted from placenta, as described earlier, was assessed with a commercially available MethylFlash ™ global DNA methylation (5-mC) and hydroxymethylation (5-hmC) ELISA Kit Epigentek (Farmingdale, NY, USA). Briefly, purified sample DNA (100 ng) in replicates was incubated in strip wells with binding solution to promote adherence to the bottom of a well. Sample wells were treated with diluted 5-mC or 5-hmC to capture and detect antibodies to measure DNA methylation status, which was quantified colorimetrically by absorbance readings at 450 nm using the VICTOR3 Multilabel Plate Reader (PerkinElmer, Shelton, USA). Both negative and positive DNA controls were provided in the kit, and a standard curve was constructed in the range 0.1–5% for 5-mC and 0.02–1% for 5-hmC. The percentages of methylated (5-mC%) and hydroxymethylated (5-hmC %) cytosines in total DNA were calculated using logarithmic second-order regression according to the standard curve formula. The global DNA methylation status was presented as the ratio of 5-mC% to 5-hmC%.

### 4.5. Statistical Analysis

The data were analyzed using STATISTICA 13 software, StatSoft, Tulsa, OK, USA. The median (Me) and interquartile range (IQR) were estimated for continuous variables, whereas absolute numbers (*n*) and percentages (%) were estimated for categorical variables.

Due to the small sizes of subgroups, we used the following nonparametric statistical tests:

- The Kruskal–Wallis H test for the comparison of continuous variables among the three modes of birth;
- Fisher’s exact test for the comparison of categorical variables among the three modes of birth;
- The Mann–Whitney U test for the comparison of 5-mC/5-hmC ratio in the placenta and *ADIPOQ* and *LEP* between two modes of birth in pairs, between genders, or for epidural analgesia versus none;
- Spearman’s correlation coefficient *r* for the correlation of 5-mC/5-hmC ratio in the placenta, *ADIPOQ* vs. *LEP*, 5-mC/5-hmC ratio in the placenta, and *ADIPOQ*/*LEP* with numerical maternal or offspring outcomes.

All correlations were estimated in the total study group, as well as separately, in every mode of birth.

The significance level was assumed at 0.05 in all statistical tests.

## 5. Conclusions

The methylation of a single gene might be different depending on the mode of delivery and uterine contractions. Further studies should concentrate on locus-specific analysis of the whole genome to detect the methylation index of specific genes involved in metabolism. Moreover, it is necessary to compare the methylation status of selected genes with the protein level in the placenta, as well as in the maternal and fetal serum. Regarding the epigenetic modulation in obstetrics, research in this field is important, since the cesarean section rate will continue to rise. More studies with a higher number of participants and follow-up should be performed.

## Figures and Tables

**Table 1:** Study group clinical characteristics.

Variable, Parameter	Unit or Category	Total Group (*N* = 70)	Elective CS (*N* = 30)	Intrapartum CS (*N* = 13)	Vaginal Delivery (*N* = 27)	*p* ^1^
Maternal age, Me (IQR)	years	32 (28–35)	34 (31–37)	29 (27–34)	31 (28–33)	**0.036**
Gestational age (weeks), *n* (%)	37	6 (8.57)	2 (6.67)	1 (7.69)	3 (11.11)	**0.026**
	38	11 (15.71)	5 (16.67)	2 (15.38)	4 (14.81)	
	39	32 (45.71)	20 (66.67)	4 (30.77)	8 (29.63)	
	40	9 (12.86)	1 (3.33)	1 (7.69)	7 (25.93)	
	41	12 (17.14)	2 (6.67)	5 (38.46)	5 (18.52)	
Parity, *n* (%)	1	38 (54.29)	13 (43.33)	11 (84.62)	14 (51.85)	0.092
	2	29 (41.43)	16 (53.33)	2 (15.38)	11 (40.74)	
	3	3 (4.29)	1 (3.33)	0 (0.00)	2 (7.41)	
Pre-gestational BMI, Me (IQR)	kg/m^2^	22.89 (20.82–25.01)	23.58 (20.90–25.46)	21.48 (19.47–22.31)	22.86 (21.26–24.91)	0.127
Intragestational BMI, Me (IQR)	kg/m^2^	28.49 (25.95–30.80)	29.10 (26.93–30.85)	25.95 (25.71–29.30)	28.26 (26.64–30.45)	0.476
Intragestational weight gain, Me (IQR)	kg	16 (11–19)	16 (12–20)	17 (14–19)	15 (11–18)	0.551
	%	25.00 (18.33–31.75)	24.23 (19.05–28.17)	32.08 (25.00–33.93)	22.22 (16.92–29.23)	0.129
Newborn weight, Me (IQR)	kg	3.58 (3.30–3.85)	3.53 (3.32–3.90)	3.60 (3.45–3.80)	3.65 (3.30–3.90)	0.962
Newborn gender, *n* (%)	male	32 (45.71)	13 (43.33)	8 (61.54)	11 (40.74)	0.470
	female	38 (54.29)	17 (56.67)	5 (38.46)	16 (59.26)	
1 min Apgar score, *n* (%)	8	2 (2.86)	1 (3.33)	1 (7.69)	0 (0.00)	0.066
	9	10 (14.29)	7 (23.33)	2 (15.38)	1 (3.70)	
	10	58 (82.86)	22 (73.33)	10 (76.92)	26 (96.30)	
5 min Apgar score, *n* (%)	9	3 (4.29)	2 (6.67)	0 (0.00)	1 (3.70)	0.999
	10	67 (95.71)	28 (93.33)	13 (100.00)	26 (96.30)	
Duration of uterine contraction ^2^, Me (IQR)	hours	-	-	-	5.17 (3.42–7.67)	-
Analgesia, *n* (%)	spinal for cesarean section or epidural for vaginal delivery	59 (84.29)	30 (100.00)	13 (100.00)	16 (59.26)	-

Me—median, IQR—interquartile range (25–75%), CS—cesarean section, BMI—body mass index. ^1^ Kruskal–Wallis’ H test or Fisher’s exact test was used to compare continuous or categorical variables, respectively, among three modes of birth. ^2^ Only in vaginal deliveries. Significant differences are in bold.

**Table 2:** Correlations between 5-mC/5-hmC ratio in the placenta and maternal or offspring outcomes.

Variable	Method	Total Group (*N* = 70)		Elective CS (*N* = 30)		Intrapartum CS (*N* = 13)		Vaginal Delivery (*N* = 27)	
		Test	*p*	Test	*p*	Test	*p*	Test	*p*
Maternal age (years)	*r*	−0.008	0.946	0.347	0.060	−0.434	0.139	0.172	0.390
Gestational age (weeks)	*r*	0.105	0.388	0.028	0.88	0.020	0.948	0.096	0.634
Parity	*r*	−0.133	0.273	0.196	0.299	−0.114	0.711	−0.308	0.118
Pregestational BMI (kg/m^2^)	*r*	−0.084	0.491	0.206	0.275	−0.121	0.694	−0.233	0.243
Intragestational BMI (kg/m)	*r*	0.047	0.701	0.317	0.088	0.143	0.641	−0.162	0.421
Intragestational weight gain (kg)	*r*	0.238	**0.047**	0.122	0.522	0.520	0.069	0.096	0.632
Intragestational weight gain (%)	*r*	0.212	0.078	−0.034	0.858	0.512	0.074	0.150	0.455
Newborn weight (kg)	*r*	0.093	0.444	0.355	0.054	−0.174	0.571	0.015	0.940
Newborn gender	*Z*	−1.438	0.150	−0.942	0.346	−2.196	**0.028**	0.444	0.657
1 min Apgar score	*r*	−0.112	0.357	−0.271	0.147	0.056	0.856	−0.050	0.803
5 min Apgar score	*r*	−0.072	0.556	−0.216	0.251	na	-	−0.050	0.803
Epidural analgesia ^1^	*Z*	-	-	-	-	-	-	−0.493	0.622
Uterine contraction (duration in hours) ^1^	*r*	-	-	-	-	-	-	0.160	0.426

*r*—Spearman’s correlation coefficient, *Z*—the Mann–Whitney U test. ^1^ Only in vaginal deliveries; CS—cesarean section. na—not applicable, as all newborns delivered via intrapartum CS had the same 5 min Apgar score at 10. Significant correlations are in bold.

**Table 3:** Primers used in the methylation-specific real-time qPCR assay. Primer sequences were designed specifically using methylated and unmethylated templates after bisulfite modification. The size of the PCR product, annealing temperature (T_a_) for primer pairs, the number of CpG sites that are within the PCR product, reference sequence, and location of the amplified region are shown.

Target Gene	Target CpG Status	Primer Name	Primer Sequence	Product Size (bp)	Ta (°C)	Number of CpG Sites	Reference: Amplified Region
*ADIPOQ*	Methylated	ADIPOQ_M3 ADIPOQ_M4	5′–TATTTTATATGATTATATTTCGCGG 5′–AACTCTATTCTAACTTCCTAACGAA	121	57	6	NG_021140.1: 8205..8327 nt
	Unmethylated	ADIPOQ_U3 ADIPOQ_U4	5′–TTTATTTTATATGATTATATTTTGTGG 5′–AACTCTATTCTAACTTCCTAACAAA	123	57		
*LEP*	Methylated	LEP_M1 LEP_M2	5′–TAGGATTAACGAGGGCGTAGTC 5′–AACCCCTTAAAAAAATACTTCGAA	111	60	14	NG_007450.1: 4586..4698 nt
	Unmethylated	LEP_U1 LEP_U2	5′–TTAGGATTAATGAGGGTGTAGTTGT 5′–CAACCCCTTAAAAAAATACTTCAAA	113	60		

**Table 4:** Correlations between *ADIPOQ* demethylation index and maternal or neonatal outcomes.

Variable	Method	Total Group (*N* = 70)		Elective CS (*N* = 30)		Intrapartum CS (*N* = 13)		Vaginal Delivery (*N* = 27)	
		Test	*p*	Test	*p*	Test	*p*	Test	*p*
Maternal age (years)	*r*	−0.115	0.340	−0.216	0.251	−0.041	0.893	0.309	0.117
Gestational age (weeks)	*r*	0.002	0.988	−0.145	0.445	0.316	0.293	−0.113	0.575
Parity	*r*	−0.240	**0.044**	−0.212	0.262	0.342	0.253	−0.249	0.211
Pregestational BMI (kg/m^2^)	*r*	−0.080	0.516	−0.270	0.149	0.385	0.194	0.085	0.672
Intragestational BMI (kg/m)	*r*	0.021	0.855	−0.195	0.303	0.523	0.067	0.192	0.336
Intragestational weight gain (kg)	*r*	0.200	0.097	0.221	0.240	0.190	0.534	0.172	0.390
Intragestational weight gain (%)	*r*	0.177	0.142	0.193	0.308	0.066	0.830	0.162	0.421
Newborn weight (kg)	*r*	−0.009	0.944	0.186	0.324	0.135	0.660	−0.114	0.571
Newborn gender	*Z*	1.002	0.316	0.146	0.884	0.001	0.999	2.245	**0.025**
1 min Apgar score	*r*	0.033	0.087	0.242	0.198	−0.268	0.376	0.101	0.617
5 min Apgar score	*r*	0.182	0.133	0.208	0.269	na	-	0.101	0.617
Epidural analgesia ^1^	*Z*	-	-	-	-	-	-	1.900	0.057
Uterine contraction (duration in hours) ^1^	*r*	-	-	-	-	-	-	0.307	0.119

*r*—Spearman’s correlation coefficient, *Z*—the Mann–Whitney U test. ^1^ Only in vaginal deliveries; CS—cesarean section. na—not applicable as all newborns delivered via intrapartum CS had the same 5 min Apgar score of 10. Significant correlations are in bold.

**Table 5:** Correlations between *LEP* demethylation index and maternal or neonatal outcomes.

Variable	Method	Total Group (*N* = 70)		Elective CS (*N* = 30)		Intrapartum CS (*N* = 13)		Vaginal Delivery (*N* = 27)	
		Test	*p*	Test	*p*	Test	*p*	Test	*p*
Maternal age (years)	*r*	−0.059	0.625	−0.090	0.635	−0.036	0.907	0.022	0.913
Gestational age (weeks)	*r*	−0.056	0.644	0.035	0.855	−0.270	0.372	0.026	0.899
Parity	*r*	0.294	**0.013**	−0.024	0.899	−0.228	0.454	0.606	**0.001**
Pregestational BMI (kg/m^2^)	*r*	−0.103	0.397	−0.347	0.060	0.267	0.378	−0.306	0.120
Intragestational BMI (kg/m)	*r*	−0.104	0.392	−0.307	0.099	0.294	0.329	−0.183	0.360
Intragestational weight gain (kg)	*r*	0.020	0.867	0.264	0.158	−0.218	0.474	−0.013	0.948
Intragestational weight gain (%)	*r*	0.022	0.854	0.276	0.140	−0.305	0.310	0.143	0.476
Newborn weight (kg)	*r*	0.087	0.474	0.022	0.910	0.171	0.577	0.140	0.485
Newborn gender	*Z*	0.318	0.750	0.167	0.867	0.000	1.000	−0.419	0.675
1 min Apgar score	*r*	0.047	0.702	0.056	0.768	0.179	0.559	−0.252	0.205
5 min Apgar score	*r*	0.063	0.605	0.309	0.097	na	-	−0.252	0.205
Epidural analgesia ^1^	*Z*	-	-	-	-	-	-	−2.245	**0.025**
Uterine contraction (duration in hours) ^1^	*r*	-	-	-	-	-	-	−0.395	**0.041**

*r*—Spearman’s correlation coefficient, *Z*—Mann–Whitney U test. ^1^ Only in vaginal deliveries; CS—cesarean section. na—not applicable as all newborns delivered via intrapartum CS had the same 5 min Apgar score of 10. Significant correlations are in bold.

**Table 6:** Correlations between 5-mC/5-hmC ratio in the placenta and *ADIPOQ* and *LEP* demethylation index in the study group.

Variable	Total Group (*N* = 70)		Elective CS (*N* = 30)		Intrapartum CS (*N* = 13)		Vaginal Delivery (*N* = 27)	
	*r*	*p*	*r*	*p*	*r*	*p*	*r*	*p*
5-mC/5-hmC ratio in the placenta and *ADIPOQ*	0.144	0.235	0.147	0.437	−0.236	0.437	0.190	0.343
5-mC/5-hmC ratio in the placenta and *LEP*	−0.024	0.841	0.072	0.706	−0.077	0.803	0.004	0.983
*ADIPOQ* and *LEP*	−0.156	0.198	0.226	0.230	−0.027	0.929	−0.456	**0.017**

## Data Availability

The data and materials used in the current study are available from the corresponding author on reasonable request.
